# Technology anxiety and resistance to change behavioral study of a wearable cardiac warming system using an extended TAM for older adults

**DOI:** 10.1371/journal.pone.0227270

**Published:** 2020-01-13

**Authors:** Tsai-Hsuan Tsai, Wen-Yen Lin, Yung-Sheng Chang, Po-Cheng Chang, Ming-Yih Lee

**Affiliations:** 1 Department of Industrial Design, College of Management, Chang Gung University, Taoyuan, Taiwan; 2 AI Innovation Research Center, Chang Gung University, Taoyuan, Taiwan; 3 Division of Cardiology, Department of Internal Medicine, Chang Gung Memorial Hospital, Linkou, Taoyuan, Taiwan; 4 Department of Visual Communication Design, Ming Chi University, New Taipei City, Taiwan; 5 Department of Electrical Engineering, Center for Biomedical Engineering, Chang Gung University, Taoyuan, Taiwan; 6 Division of Cardiology, Department of Internal Medicine, Chang Gung Memorial Hospital, Linkou, Taoyuan, Taiwan; 7 School of Information, The University of Texas at Austin, Austin, Texas, United States of America; 8 Chang Gung Memorial Hospital, Linkou Medical Center, Taoyuan, Taiwan; 9 Graduate Institute of Medical Mechatronics, Center for Biomedical Engineering, Chang Gung University, Taoyuan, Taiwan; Universiteit Twente, NETHERLANDS

## Abstract

With advances in technology, wireless and sensor technologies represent a method for continuously recording people’s biomedical signals, which may enhance the diagnosis and treatment of users’ everyday health conditions. These technologies mostly target older adults. In this study, we examine a smart clothing system targeting clinically high-risk patients, including older adults with cardiovascular disease (31 outpatients) and older adults in general (81 participants), to obtain an understanding of the patients’ perception of using wearable healthcare technologies. Given that technology anxiety has been shown to affect users’ resistance to using new technology and that perceived ubiquity is considered a characteristic of wearable devices and other mobile wireless technologies, we included three external variables: i.e., technology anxiety, perceived ubiquity, and resistance to change, in addition to the traditional components of the technology acceptance model (TAM). The results of the hypothesized model showed that among older adults in general, technology anxiety had a negative effect on the perceived ease of use and perceived ubiquity. The perceived ubiquity construct affects both user groups’ perceived ease of use and perceived usefulness of wearing smart clothes. Most relationships among the original constructs of the TAM were validated in older adults in general. Interestingly, we found that perceived usefulness had an indirect effect on behavioral intention through attitude. These *results* further confirm the validity of the extended TAM in determining older users’ technology acceptance behavior.

## Introduction

With improvements in sanitation, enhanced living standards, medical advances, and the development of technologies, the average life expectancy has increased globally over the past few decades, especially in developed countries [1]. For example, the percentage of the elderly population (aged ≥ 65 years) in Japan is expected to exceed 30% in 2025 and to reach 39.9% in 2060, while the national total population reached its peak in 2008 and has subsequently decreased steadily [2]. The population demographics in Taiwan have also exhibited a dramatic change over the past few years. The percentage of older adults in Taiwan was only approximately 2.5% in the mid-20^th^ century, but with rapid industrialization and modernization, Taiwan became an aging society in 1993, with older adults accounting for 7% of the population. Taiwan is expected to become a super-aged society (20% of the population aged above 65) by approximately 2025 [3]. A similar phenomenon has been observed in Western countries; for example, the older adult population in the United States is expected to nearly double from 48 million to 88 million by 2050 [4]. Although increased life expectancy can be considered an indication of positive human and societal development, it leads to challenges in older adults’ health conditions.

As people age, their physical and mental health conditions may begin to degenerate. This degeneration is a major cause of chronic diseases, such as heart disease, stroke, cancer, and type 2 diabetes. Among all types of chronic diseases, heart disease or cardiovascular disease, which is a general term used to describe a group of disorders of the heart and blood vessels, is the leading cause of death worldwide [5]. Of the 56.4 million deaths globally in 2015, more than 8 million were due to heart disease. Heart disease also caused the largest number of deaths in the United States from 1980 to 2014, with 614,348 deaths due to heart disease in 2014 [6]. Heart disease is more likely to occur with aging. Only 1% of atrial fibrillation patients are aged under 60 years, while more than one-third of these patients are older than 80 years [7, 8].

When patients visit a clinic for routine health examinations, they are expected to report any heart-related symptoms since their prior visit. However, without an immediate or real-time record of cardiac activity, patients’ self-reports may be biased, inaccurate, or incomplete. Given the high prevalence of heart disease worldwide, especially in the older adult population, and the need for accurate outpatient information regarding heart conditions, many wireless and sensor technologies, such as finger-ring sensors [9], smartwatches [10] and smartphone applications [11, 12], have been developed to assist patients in understanding and managing their heart conditions in their daily lives. For instance, Athilingam et al. [12] developed a mobile application for heart failure patients to record their heart rate, heart rate variability and respiratory rate using a Bluetooth sensor worn on the chest. The features of the app include a health assessment, exercise information, real-time heart vital signs, and chronic heart failure information.

Wireless and sensor technologies provide a method for continuously recording people’s biomedical signals (e.g., heart rate), which may enhance the diagnosis and treatment of users’ everyday health conditions. Furthermore, given their ubiquitous ability to detect daily biomedical signals, wireless and sensor technologies may replace parts of medical devices that are available only in medical institutions. This development could facilitate medical care for patients and decrease the overutilization of medical resources, enabling people in need to benefit from medical care. The design of wireless and sensor technologies could also integrate multiple biomedical signal detections into a single wearable technology rather than requiring multiple technologies with various functions. Heart disease detection at an early stage could significantly decrease heart disease mortality and supplement inadequate detection by medical institutions. For example, certain abnormal heart conditions may not be detected when patients visit a clinic for their regular heart check. Hence, the use of wearable technologies can increase patients’ daily safety and can be used to detect abnormal heart conditions [13].

Currently, most wearable technologies that can detect heart conditions are designed for sports use, health management, or specific types of medical examinations. Based on the system needs for different types of biomedical signals, there are various design considerations for different types of products and wearing styles. The Amiigo fitness bracelet is designed primarily to detect a user’s exercising conditions. When the bracelet detects that the user is exercising, the device begins to track the user’s heart rate, blood pressure, oxygen saturation, and calories burned [14]. Zio XT, which is a water-resistant, wire-free patch attached to the user’s upper-left chest, can capture beat-to-beat cardiac rhythms over 14 continuous days and detect infrequent or asymptomatic arrhythmias. The design of the monitor makes it easy to wear and use without interfering in daily activities and sleep [15]. QardioCore is another device that integrates an ECG monitor with a smartphone app, enabling users to detect their heart condition. The device, which is designed specifically for medical care and is suitable for people with increased health risks, can automatically share a patient’s personal data with doctors. Users wear the product, which has four sensors, near their lower chest [16].

In addition to the above-mentioned types of devices, the implementation of wireless and sensor technologies for heart detection through the design of “smart clothing” could help in detecting patients’ health conditions. Lin, Chou, Tsai, Lin, and Lee [17] developed a novel, textile-based, intelligent wearable instrumented vest for posture monitoring with multichannel accelerometer-based motion-sensing technologies. This garment can process various information, such as the tilting angles of the sensors and internal event detection. In particular, the interactive real-time posture-monitoring software developed for the vest can be used in various healthcare applications. Researchers have focused on developing smart clothing for patients with cardiovascular disease. The VivoMetrics LifeShirt was one of the earliest smart clothing items made in the United States and can measure users’ heart and pulmonary parameters [18]. The Wearable Health Care System (WEALTHY) project, which was supported by the European Commission, designed a system to target clinically high-risk patients with chronic diseases. Electrocardiography (ECG) sensors are integrated into the fabric to monitor the respiratory rate, body position and movement [19]. Another project supported by the European Commission and industrial partners is the MyHeart project. The MyHeart system consists of an ECG and activity sensor embedded in the garment to diagnose cardiovascular disease [20].

Many wearable devices and products have been designed to specifically measure cardiovascular disease patients’ heart conditions. However, although most cardiovascular disease patients are older adults, the design of existing wearable technologies is not based on older adults’ needs and considerations. Older adults must continuously monitor and diagnose their heart conditions regardless of whether they have been previously diagnosed with cardiovascular disease. Furthermore, unique factors, such as technology anxiety, could affect older adults’ willingness to use and acceptance of using wearable technologies [21]. In terms of academic research, studies exploring the acceptance of using wearable technologies among older adults with chronic diseases and older adults without chronic diseases are lacking. Thus, in the current research, we use smart clothing as an example to understand older adults’ willingness to accept wearable technology. The technology acceptance model (TAM) is used to examine the perceptions of two groups of older adults regarding the use of wearable technologies in the healthcare domain.

## Hypothesis development

**Fig 1:**
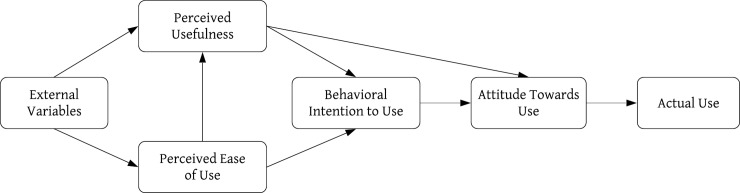
Technology acceptance model [23].

The TAM, which was adapted from the theory of reasoned action [22], was proposed by Davis [23]. The purpose of the model is to explain people’s acceptance of technology. This model explains the relationship between external variables, which are a system’s characteristics, and the likelihood that a user will use a system, which is measured by the user’s attitude and behavioral intention [24]. According to the TAM, the use and selection of new technology are determined by the behavioral intention of using the technology. Perceived ease of use (PEOU) and perceived usefulness (PU) are the two key psychological constructs that determine users’ attitudes toward the use of a technology or service. If a technology is perceived as easy to use and useful in accomplishing a task, then users will have a more positive attitude toward the technology. Furthermore, a user’s attitude subsequently affects his/her intention to actually use the technology. The external variables of a technology could affect the perceived ease of use and perceived usefulness of a technology [25]. PEOU may also directly influence PU, and PU may directly influence the behavioral intention to use, which in turn may determine whether a user actually uses a technology [23]. However, it is also important to understand how external variables affect the PEOU and PU [26]. The original TAM is shown in Fig 1.

In recent years, there has been an increase in research attention focusing on users’ perceptions of wearable technologies [17, 27–29]. Among these studies, the TAM is one of the most extensively used models to study an individual’s acceptance of information and communication technologies [23]. For example, Chuah et al. [28] utilized this model to explain why business students are willing to adopt smartwatches. In addition to the original constructs in the TAM, Chuah et al. [28] investigated the effect of visibility, or the extent to which users believe that other people noticed them wearing smartwatches. The results showed that users perceived smartwatches as both technology and fashion statements. Furthermore, visibility and perceived usefulness can affect users’ attitudes and intentions in using smartwatches. Kim and Shin [30] used the TAM, integrated with other psychological constructs such as affective quality, relative advantage, mobility, availability, and subcultural appeal, to understand users’ adoption of smartwatches. Their model revealed that smartwatches are perceived as easy to use if they have high mobility and availability, and those with greater affective quality and relative advantage are viewed as more useful. Lunney et al. [29] used the TAM to understand MTurk workers’ perceptions of a wearable fitness technology and investigated the relationship between the use of wearable fitness technologies and perceived health benefits. The results showed that wearable devices that are perceived as easy to use and useful are more likely to be adopted by consumers. In addition, a significant positive relationship has been found between the use of wearable fitness technologies and perceived health benefits. Correspondingly, some researchers have studied users’ acceptance of smart clothing technologies. Schaar and Ziefle [31] asked 100 mostly healthy participants to indicate whether the expected benefits or the perceived shortcomings of smart clothing would affect their overall acceptance. The participants completed a questionnaire regarding the pros and cons of using smart clothes, and the results showed that gender and technical experience affected users’ acceptance of the use of smart clothes such that women and people with low technical experience were more reluctant to use this technology. Spagnolli, Guardigli, Orso, Varotto, and Gamberini [32] asked either general or professional users (firefighters, volleyball players, elderly people, or neuroscientists) to assess their acceptance of wearable devices by answering a questionnaire that partially included factors of the TAM. Perceived usefulness, perceived comfort, facilitating conditions, and attitude toward the technology were found to be good predictors of users’ acceptance. Compared with young adults, elderly people tend to be anxious about using unfamiliar technology. Lin et al. [17] extended the TAM by adding technology anxiety as an external variable to examine 50 elderly people’s perceptions of a newly designed posture-monitoring vest. The results indicated that technology anxiety negatively affected older adults’ perceived ease of use of smart clothes. Notably, although elderly people expressed anxious feelings toward the use of smart clothes, they still exhibited a positive attitude toward the use of smart clothes in the future. Although the TAM has been used successfully to explain perceptions of wearable technologies, recent studies have primarily focused on wearable devices rather than smart clothing. In addition, participants in previous studies were generally healthy rather than older or with cardiovascular disease. Thus, this study proposes an extension of the original TAM by adding external variables. The goal is to examine the effects of external factors on attitudes and behavioral intentions and the decision to use smart clothing technology among older people as users, potential users and cardiac patients.

Because wearable technology is a newly developed and widely adapted technology, it is important for researchers to understand users’ intention to use wearable devices. Over the past few years, studies investigating wearable devices have focused on the health purpose of the products and have used the TAM to explain users’ intention to use the products [33]. Nevertheless, the design of the products did not consider “age” as a significant factor that could affect potential users’ intention to use wearable devices. Thus, in this study, we extend the TAM to obtain an understanding of how other external constructs, such as technology anxiety, perceived ubiquity, and resistance to change, affect older adults’ perceptions of the use of wearable technology in the healthcare domain.

### Technology anxiety

The term technology anxiety is derived from early studies of computer anxiety [34]. Although the early development of the computer showed significant promise in simplifying people’s lives and work, numerous researchers were interested in the psychological characteristics of naïve computer users that led them to hold anxious and negative attitudes toward computers [35, 36]. Some of the factors found to cause computer anxiety include previous computer experience, age, gender, self-efficacy, and mathematic anxiety [37]. For example, without sufficient experience with computers, users are afraid that operating a computer will lead to uncorrectable mistakes.

Computer anxiety can affect users’ perceptions of computers. Igbaria et al. [38] found that computer anxiety negatively affects a person’s computer usage, perceived enjoyment, and perceived usefulness. In a systematic literature review, anxiety was identified as a factor that affected patients’ acceptance of consumer health information technology. Or and Karsh [39] found that fear of technology was negatively associated with patients’ acceptance of health technology. In addition, their results revealed that older adults were more likely to experience anxiety and less comfort and control when using technologies; this finding has been further confirmed in related studies [21, 40]. Many studies have confirmed the negative relationship between technology anxiety and perceived ease of use [21, 40]. For example, Chang and Im [40] found that older adults’ lack of computer experience was related to their greater computer anxiety; thus, accessing health information on the Internet is more difficult for older adults. Furthermore, among the elderly in China, technology anxiety was found to be negatively related to the PEOU of using mobile health services [21]. Additionally, because cardiovascular disease patients are more concerned about their health condition than are older adults in general, they may be more anxious and less likely to trust smart clothing technologies. Thus, in this study, we postulated the following hypotheses:

**H1a.** Technology anxiety is negatively associated with the perceived usefulness of smart clothing among older adults with cardiovascular disease.
**H1b.** Technology anxiety is negatively associated with the perceived usefulness of smart clothing among older adults in general.
**H2a.** Technology anxiety is negatively associated with the perceived ease of use of smart clothing among older adults with cardiovascular disease.
**H2b.** Technology anxiety is negatively associated with the perceived ease of use of smart clothing among older adults in general.
**H3a.** Technology anxiety is positively associated with resistance to change regarding smart clothing among older adults with cardiovascular disease.
**H3b.** Technology anxiety is positively associated with resistance to change regarding smart clothing among older adults in general.
**H4a.** Technology anxiety is negatively associated with the perceived ubiquity of smart clothing among older adults with cardiovascular disease.
**H4b.** Technology anxiety is negatively associated with the perceived ubiquity of smart clothing among older adults in general.

### Resistance to change

Currently, the introduction of smart clothes is not widely known or accepted among potential users. When users are faced with a new service, they may be reluctant to switch from one service to an alternative one. According to Bhattacherjee and Hikmet [41], resistance to change is defined as “a generalized opposition to change engendered by the expected adverse consequences of change”. Previous research has examined the relationship between technology anxiety and resistance to change [21, 34] and has shown that technology anxiety can lead to resistance to change due to uncertainty and unexpected errors of technology [42]. Guo et al. [21] suggested that older adults were more reluctant to use mobile health services if they reported having higher technology anxiety. Furthermore, users’ sensitivity and high levels of resistance to using new technology may affect their evaluation regarding the usefulness of the technology. Guo et al. [21] also found that resistance to change negatively affects older adults’ perception of the usefulness of mobile health services. Thus, in this study, we postulated the following:

**H5a.** Resistance to change is negatively associated with the perceived usefulness of smart clothing among older adults with cardiovascular disease.
**H5b.** Resistance to change is negatively associated with the perceived usefulness of smart clothing among older adults in general.
**H6a.** Resistance to change is negatively associated with the perceived ease of use of smart clothing among older adults with cardiovascular disease.
**H6b.** Resistance to change is negatively associated with the perceived ease of use of smart clothing among older adults in general.

### Perceived ubiquity

Because of advancements in technology, people have the luxury of accessing information anytime and anywhere through mobile devices. Similarly, one characteristic of wearable devices and other mobile wireless technologies is mobility or perceived ubiquity. With the extended use of batteries and wireless sensors, users’ physiological signals can be detected and recorded ubiquitously. Kim and Garrison [43] defined perceived ubiquity as an “individual’s perception regarding the extent to which mobile wireless technology provides personalized and uninterrupted connection and communications between the individual and other individuals and/or networks”. In the current study, perceived ubiquity reflects a patient’s perception of the extent to which smart clothing technology can receive and record his or her health condition anytime and anywhere.

Perceived ubiquity has been found to have a positive effect on perceived ease of use. Kim and Shin [30] emphasized that users judge smartwatches as easier to use because of their mobility. Hsiao and Tang [44] investigated users’ acceptance of mobile healthcare technology among older adults in Taiwan and found that perceived ubiquity and perceived ease of use affected these older adults’ attitudes toward wearing mobile healthcare technology. Thus, in this study, we postulated the following:

**H7a.** Perceived ubiquity is positively associated with the perceived usefulness of smart clothing among older adults with cardiovascular disease.
**H7b.** Perceived ubiquity is positively associated with the perceived usefulness of smart clothing among older adults in general.
**H8a.** Perceived ubiquity is positively associated with the perceived ease of use of smart clothing among older adults with cardiovascular disease.
**H8b.** Perceived ubiquity is positively associated with the perceived ease of use of smart clothing among older adults in general.

### Perceived usefulness, perceived ease of use, and attitude

As mentioned previously, perceived usefulness and perceived ease of use are two cognitive belief constructs that affect users’ attitudes and that subsequently determine users’ intention to use. The relationships among these four constructs have been firmly established in various studies using the TAM, in which PEOU has been shown to have positive relationships with PU and attitude and PU has been shown to have a positive relationship with attitude. Attitude is also positively related to behavioral intention. According to the definition proposed by Davis [23], perceived usefulness is “the extent to which a person believes that using a particular technology will enhance his/her job performance”, whereas perceived ease of use is defined as “the degree to which a person believes that using a technology will be free from effort”. Although the definition of perceived usefulness specifically mentions “job performance”, the TAM has been generalized in different fields, and perceived usefulness has been described as how useful a technology can be in accomplishing a goal.

In recent studies that used the TAM to investigate users’ perceptions of wearable technologies, Lunney et al. [29] and Chuah et al. [28] posited that users have a more positive attitude toward wearable technologies if the technologies are perceived as useful. Chuah et al. [28] further found that users perceive wearable technologies as more useful if the technologies are easy to use. Lin et al. [17] indicated that older adults had a more positive attitude toward a wearable instrumented vest if it was perceived as easy to use. Finally, both Chuah et al. [28] and Lin et al. [17] demonstrated that if users have positive attitudes toward wearable technologies, they have a greater intention to use these technologies. Thus, we formulated the following hypotheses:

**H9a.** Perceived usefulness is positively associated with attitudes toward smart clothing among older adults with cardiovascular disease.
**H9b.** Perceived usefulness is positively associated with attitudes toward smart clothing among older adults in general.
**H10a.** Perceived usefulness is negatively associated with the behavioral intention to use smart clothing among older adults with cardiovascular disease.
**H10b.** Perceived usefulness is positively associated with the behavioral intention to use smart clothing among older adults in general.
**H11a.** Perceived ease of use is positively associated with attitudes toward smart clothing among older adults with cardiovascular disease.
**H11b.** Perceived ease of use is positively associated with attitudes toward smart clothing among older adults in general.
**H12a.** Perceived ease of use is positively associated with the perceived usefulness of smart clothing among older adults with cardiovascular disease.
**H12b.** Perceived ease of use is positively associated with the perceived usefulness of smart clothing among older adults in general.
**H13a.** Attitude is positively associated with the behavioral intention to use smart clothing among older adults with cardiovascular disease.
**H13b.** Attitude is positively associated with the behavioral intention to use smart clothing among older adults in general.

## Methods

All participants and researchers were protected and restricted by the Institutional Review Board (IRB). Ethical approval was obtained from the Institutional Review Board of Chang Gung Hospital, Taoyuan, Taiwan (104-8175B). All relevant ethical safeguards were met relative to ethical considerations and subject protection.

### Participants

In total, 81 participants, including 50 older adults residing at Chang Gung Health and Culture Village and 31 older adults with cardiovascular disease recruited from Chang Gung Memorial Hospital, Taoyuan, Taiwan, agreed to participate in the study.

Among all 81 older adult participants, 53% of the participants were male and 47% of the participants were female. In terms of age, 25% of the participants were aged between 50 and 59 years; 28% of the participants were aged between 60 and 69 years; 21% of the participants were aged between 70 and 79 years; 22% of the participants were aged between 80 and 89 years; and 4% of the participants were older than 90 years. Regarding education, 8% of the participants had graduated from graduate school; 31% of the participants had graduated from a university; 20% of the participants had completed high school as their highest level of education; 10% of the participants had completed junior high school as their highest level of education; and 32% of the participants had completed elementary school as their highest level of education. None of the participants had experience wearing the smart vest. 15% of the participants had experiences wearing kneepads; 12% wore lower back support belt; and 4% wore wrist braces.

Regarding the 31 older adults with cardiovascular disease, 74% of these participants were male and 26% of these participants were female. In terms of age, 39% of the participants were aged between 50 and 59 years; 35% of the participants were aged between 60 and 69 years; 13% of the participants were aged between 70 and 79 years; 10% of the participants were aged between 80 and 89 years; and 3% of the participants were older than 90 years. Regarding education, 3% of the participants had graduated from graduate school; 29% of the participants had graduated from a university; 19% of the participants had completed high school as their highest level of education; 10% of the participants had completed junior high school as their highest level of education; and 39% of the participants had completed elementary school as their highest level of education.

The inclusion criteria for the healthy participants from Chang Gung Health and Culture Village were as follows: (a) aged over 50 years; (b) no structural heart diseases; (c) capable of reading Mandarin Chinese; and (d) a Mini-Mental State Examination (MMSE) score above 24, indicating strong mental abilities, such as memory, attention and language [45]. Regarding the elderly participants with cardiovascular disease, all qualified participants were evaluated by doctors as patients in need of smart clothing. The inclusion criteria for the participants with cardiovascular disease were as follows: (a) aged over 50 years and (b) diagnosis of either heart failure with a left ventricular ejection fraction <40% or severe valvular heart disease. All participants with cardiovascular disease were outpatients of a hospital in Northern Taiwan who visited the hospital for regular checkups with their doctors. At the end of the appointment, the doctor informed the patients of the research study and asked whether they were willing to participate. The purpose of the study and a participation fee were briefly explained to the patients. If the patients agreed to participate in the study, the nurse brought the patient to another clinical room where the researchers conducted the research survey. A similar procedure was performed with the participants from Chang Gung Health and Culture Village. The researchers explained the purpose of the study and brought the participants to the testing room if they agreed to participate in the study.

### Materials

**Fig 2:**
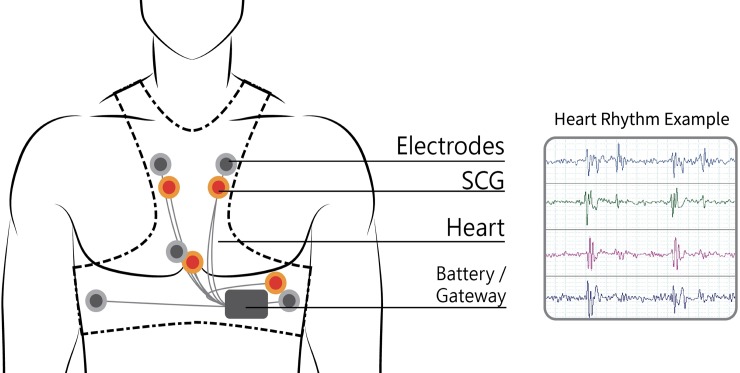
The sensor positions (gray dots: ECG sensors; red dots: SCG sensors).

**Fig 3:**
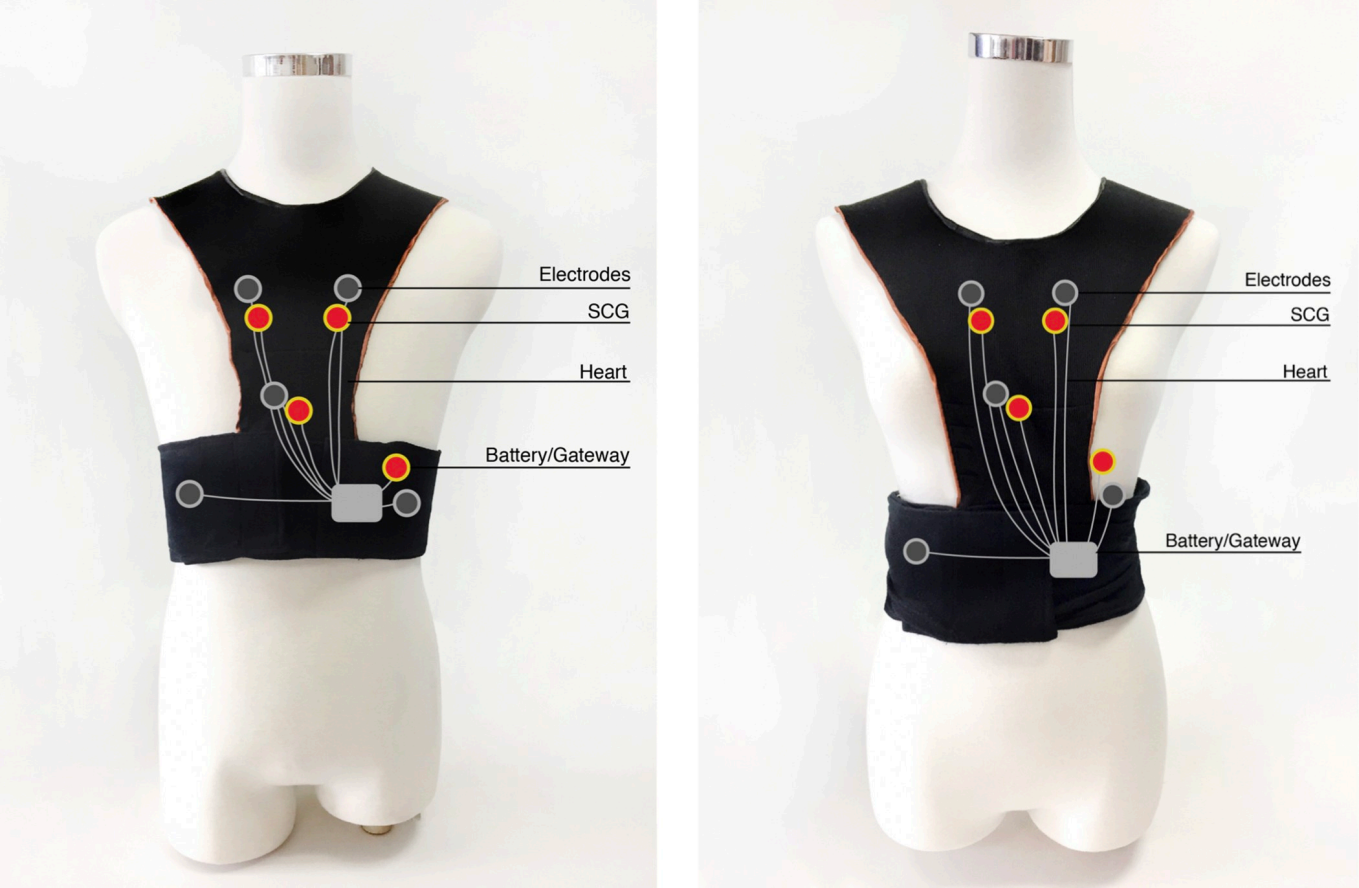
The wearable instrumented vest for males (left) and females (right).

**Fig 4:**
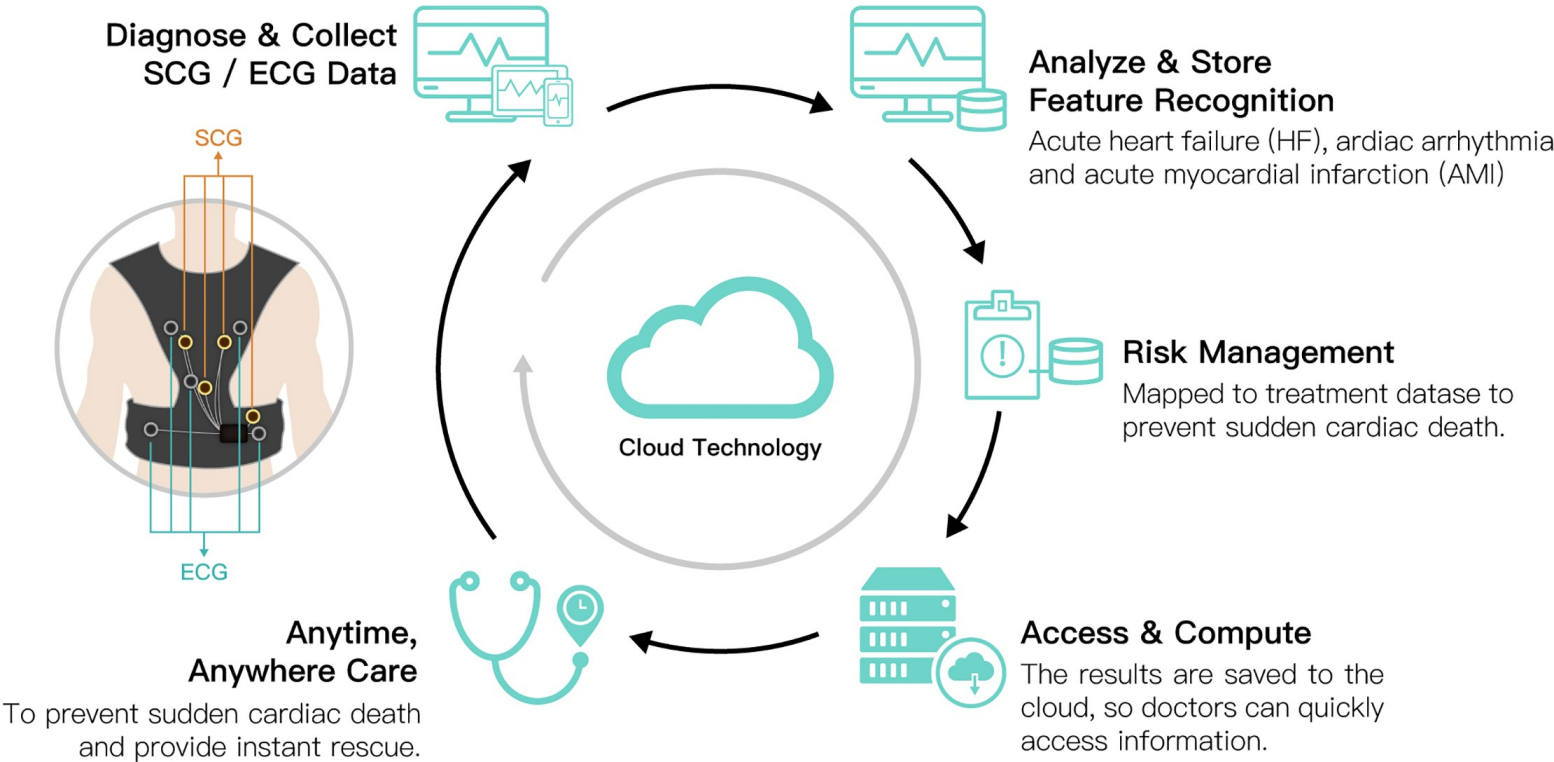
Framework of the wearable instrumented vest.

Existing smart clothing items, such as the Sensatex smart shirt, the Vivonoetics LifeShirt [46], the Fraunhofer Fitness SHIRT, and the E-Health shirt [47], monitor multiple vital signs and use only a single simple accelerometer to monitor activities or sports-related actions. As a result, they can perform only limited posture or activity detection. Conversely, Lin et al. [17] developed a wearable instrumented vest (Figs 2–4) for multi-posture monitoring to detect any harmful postures or irregular activity patterns as they occur in a timely manner to provide users with an early warning to change the posture or the pattern. Additionally, the detected data can be stored and monitored for further analysis of behavioral changes over extended periods of time. It is expected that some early predictions of the symptoms of certain chronic diseases, such as Parkinson’s disease and stroke, could be diagnosed through the long-term monitoring of critically imbalanced posture or irregular activity patterns. Based on previous achievements in wearable sensing technologies, the team of Lin et al. [17] developed a new generation of a wearable instrumented vest to perform seismocardiogram (SCG) and detect acute heart failure (HF), cardiac arrhythmia and acute myocardial infarction (AMI) and to prevent sudden cardiac death, especially in the elderly and patients with cardiovascular disease. The newly designed smart vest for cardiac activity monitoring can provide advanced, real-time, wireless, multichannel seismocardiogram front-end sensing technology with wearable sensing modules integrated with the ECG sensing module and wireless communication technology. This approach provides a gateway for ECG/SCG with online signal processing capabilities. In addition, this technology may prevent accidents associated with heart conditions from occurring and provide instant rescue for users who live alone or cannot take care of themselves when accidents occur. The newly developed smart vest was patented in Taiwan (Certification number: I603712) and received the 14th National Innovation Award from the Taiwan Institute for Biotechnology and Medicine Industry (IBMI) in 2017 and the FutureTech 2018 Award from the Taiwan Ministry of Science and Technology (MOST) for the development of the invention.

### Procedures

Upon the participants’ arrival at the testing room for the research survey, the researchers first explicitly explained the purpose and procedure of the study. Then, the participants signed the consent form. During the experimental stage, the participants’ demographic data were collected, including gender, age, education level, and experience wearing a smart vest or other wearable protection gear. Information on the experience of wearing protective gear was collected to assist the participants in imagining the use of smart clothing. Then, the participants watched a video describing the functionality of the smart clothing system developed by our research team, as shown in Fig 2 and Fig 3. The video was played on a touch pad and was 2 minutes and 15 seconds long. In the video, the researchers described the functions of the newly designed smart vest, including monitoring cardiac activity and providing advanced real-time wireless SCG and ECG sensing modules and wireless communication technology to prevent accidents associated with heart conditions from occurring and to provide instant rescue to users who live alone or cannot take care of themselves when accidents occur. Other characteristics of the newly developed smart vest, such as its fabric, benefits, and washability, were also mentioned. After watching the video, the smart vest was presented to the participants, which allowed them to actually feel the product and obtain a better sense of the texture and functionality of the wearable cardiac sensing technology. However, due to personal hygiene concerns, the participants did not wear the smart vest. In addition to presenting the video and introducing the specific features of the smart vest, a scenario was presented to the participants. The scenario described the daily lives of older adults with cardiovascular disease who wore the smart vest and how the smart vest affected their lives. For example, the smart vest can send a signal to the older adult’s smartphone to warn him/her about an irregular heart rate. After completing the scenario presentation, the participants completed a technology acceptance questionnaire. Finally, the participants were asked to verbally provide their thoughts and concerns about potentially using the smart vest. The entire survey took approximately 30 minutes. The timeline for recruiting the participants and executing the experiment was one year. The long timeline was due to difficulties in recruiting older adults with cardiovascular disease and cooperating with the doctors’ limited available time at hospitals.

### Measurement development

The questionnaire consisted of seven major sections that assessed technology anxiety, perceived ubiquity, resistance to change, perceived usefulness, perceived ease of use, attitude, and behavioral intention (Table 1). All constructs were measured using five-point Likert-type scales, with 1 indicating strongly disagree and 5 indicating strongly agree. Each construct’s corresponding questionnaire was derived and modified from a variety of sources to reflect the characteristics of smart clothes. The measures for technology anxiety and resistance to change were adapted from Guo et al. [21]. The measure for perceived ubiquity was adapted from Hsiao and Tang [44]. For perceived usefulness and perceived ease of use, the measures were derived from Davis [23]. The measure of attitude originated from Fishbein and Ajzen (22) and Ajzen (48), and the measure of behavioral intention originated from Venkatesh, Morris (49). The entire questionnaire is shown in Table 1. Before data analysis, the data were compiled in an Excel file and reviewed twice to ensure completeness and to detect errors.

**Table 1:** Measures of the constructs.

Variable	Items	Description
Technology anxiety [21]	TA1	I feel apprehensive about using the smart clothing system.
	TA2	I hesitate to use technology for fear of making mistakes that I cannot correct.
	TA3	I am afraid that the equipment may suddenly stop functioning.
	TA4	I do not want other people to see me wearing smart clothes.
Perceived ubiquity [44]	PB1	A smart clothing system that provides healthcare information “anytime and anywhere” is crucial.
	PB2	The smart clothing system provides me with anytime-and-anywhere communication and connectivity.
	PB3	I will use the smart clothing system very often for health purposes.
Resistance to change [21]	RC1	I do not want the smart clothing system to change the way I deal with health-related problems.
	RC2	I do not want the smart clothing system to change the way I keep myself healthy.
	RC3	I do not want the smart clothing system to change the way I interact with other people.
	RC4	Overall, I do not want smart clothing to change the way I currently live.
Perceived usefulness [23]	PU1	Using smart clothes will improve my life quality.
	PU2	Using the smart clothing system will make my life more convenient.
	PU3	Using the smart clothing system will make me more effective in my life.
	PU4	Overall, I find the smart clothing system to be useful in my life.
Perceived ease of use [23]	PEOU1	I find the smart clothing system to be clear and understandable.
	PEOU2	I find that the smart clothing system does not require a lot of mental effort.
	PEOU3	I find the smart clothing system to be easy to use.
Attitude [22, 48]	AT1	I think that using the smart clothing system is a good idea.
	AT2	I think that using the smart clothing system is beneficial to me.
	AT3	I have a positive perception of using the smart clothing system.
Behavioral intention [49]	BI1	I intend to use the smart clothing system in the future.
	BI2	I will always try to use the smart clothing system in my daily life.
	BI3	I plan to use the smart clothing system frequently.

## Results

Because the goal of the study was to obtain a better understanding of older adults’ acceptance of using wearable technologies, specifically smart clothing, we divided the participants into the following two potential user groups: a.) older adults with cardiovascular disease (31 participants) and b.) older adults in general (81 participants). The data analysis is based on a comparison of the two user groups.

The data analysis included the following two stages: the measurement model and structural equation modeling (SEM). SPSS 22 and Smart PLS (Partial Least Square) 3.0 were used to perform the analyses of the measurement model and SEM, respectively. The measurement model was used to test the reliability and validity of the constructs by conducting a confirmatory factor analysis. Partial least squares structural equation modeling (PLS-SEM) was used to examine each path and its hypothesis. Research has shown that PLS-SEM can be applied using a small sample size [50]. PLS-SEM is also frequently used in fields such as business, marketing strategies, and information management to analyze data distributions, sample sizes, and the use of formative indicators [51, 52]. The measurement models of both groups are explained separately, while the structural equation modeling results are discussed together.

### Measurement model of older adults with cardiovascular disease

The convergent validity and discriminant validity of the measurement model of older adults with cardiovascular disease were tested using a confirmatory factor analysis. Convergent validity tests whether the items of a construct that are expected to be related are highly correlated [53]. If Cronbach’s alpha is higher than 0.7 [54], the questionnaire is considered to have good reliability. Composite reliability (CR) greater than 0.7 [55] and average variance extracted (AVE) greater than 0.5 [55–57] are suggested for convergent validity. Table 2 shows the factor loading, Cronbach’s alpha, CR and AVE of each variable in the model of older adults with cardiovascular disease. Regarding Cronbach’s alpha, nearly all constructs had a value higher than 0.7, and only PEOU had a value of 0.692, which is close to 0.7. All measures exceeded the minimum levels, and CR ranged from 0.817 to 0.947, while AVE ranged from 0.589 to 0.855, indicating good convergent validity. The mean, standard deviation, and discriminant validity of the constructs are shown in Table 3.

**Table 2:** Reliability and validity of the measurement model of older adults with cardiovascular disease.

Variable	Item	Cronbach’s Alpha	Factor Loading	Composite Reliability	Average Variance Extracted	
**Technology Anxiety**	TA1	.792	0.89	0.847	0.589	
	TA2		0.89			
	TA3		0.67			
	TA4		0.57			
**Perceived Ubiquity**	PB1	.917	0.96	0.953	0.835	
	PB2		0.96			
	PB3		0.86			
**Resistance to Change**	RC1	.916	0.94	0.938	0.791	
	RC2		0.94			
	RC3		0.91			
	RC4		0.75			
**Perceived** **Ease of Use**	PEOU1	.692	0.79	0.817	0.598	
	PEOU2		0.79			
	PEOU3		0.75			
**Perceived Usefulness**	PU1	.934	0.93	0.953	0.835	
	PU2		0.92			
	PU3		0.88			
	PU4		0.93			
**Attitude**	AT1	.839	0.83	0.904	0.758	
	AT2		0.84			
	AT3		0.94			
**Behavior** **Intention**	BI1	.917	0.92	0.947	0.855	
	BI2		0.96			
	BI3		0.89			

**Table 3:** The mean, standard deviation, and discriminant validity of the constructs of the model of older adults with cardiovascular disease.

	Mean	S.D.	BI	AT	PU	PEOU	RC	TA	PB
**BI**	3.66	0.92	0.925						
**AT**	4.11	0.79	0.433	0.871					
**PU**	3.93	0.84	0.193	0.698	0.914				
**PEOU**	3.68	0.75	0.241	0.483	0.614	0.773			
**RC**	3.17	1.28	-0.553	-0.348	-0.286	-0.243	0.889		
**TA**	2.39	1.05	-0.211	-0.448	-0.503	-0.403	0.208	0.767	
**PB**	3.88	0.90	0.428	0.582	0.708	0.665	-0.451	-0.297	0.927

### Measurement model of older adults in general

Similar to the analysis of older adults with cardiovascular disease, the measurement model of older adults in general was tested. Table 4 shows the factor loading, Cronbach’s alpha, CR and AVE of each variable of the model for older adults in general. All measures exceeded the minimum levels. The Cronbach’s alpha values of all scales ranged from 0.717 to 0.937, the CR ranged from 0.813 to 0.958, and AVE ranged from 0.528 to 0.883, indicating good convergent validity. Thus, we concluded that the constructs had satisfactory reliability [58], and the constructs were included in further analyses. The mean, standard deviation, and discriminant validity of the constructs are shown in Table 5.

**Table 4:** Reliability and validity of the measurement model of older adults in general.

Variable	Item	Cronbach’s Alpha	Factor Loading	Composite Reliability	Average Variance Extracted
**Technology Anxiety**	TA1	.718	0.88	0.813	0.528
	TA2		0.94		
	TA3		0.82		
	TA4		0.85		
**Perceived Ubiquity**	PB1	.853	0.88	0.910	0.771
	PB2		0.88		
	PB3		0.87		
**Resistance to Change**	RC1	.902	0.88	0.928	0.765
	RC2		0.94		
	RC3		0.82		
	RC4		0.85		
**Perceived** **Ease of Use**	PEOU1	.717	0.78	0.839	0.635
	PEOU2		0.79		
	PEOU3		0.82		
**Perceived Usefulness**	PU1	.937	0.92	0.955	0.840
	PU2		0.91		
	PU3		0.93		
	PU4		0.91		
**Attitude**	AT1	.886	0.90	0.929	0.815
	AT2		0.88		
	AT3		0.93		
**Behavior** **Intention**	BI1	.934	0.92	0.958	0.883
	BI2		0.96		
	BI3		0.93		

**Table 5:** The mean, standard deviation, and discriminant validity of the constructs of the model of older adults in general.

	Mean	S.D.	BI	AT	PU	PEOU	RC	TA	PB
**BI**	3.51	0.96	0.940						
**AT**	4.06	0.80	0.670	0.903					
**PU**	3.76	0.92	0.543	0.746	0.917				
**PEOU**	3.83	0.80	0.590	0.629	0.613	0.797			
**RC**	3.43	1.18	-0.298	-0.278	-0.227	-0.171	0.875		
**TA**	2.55	1.94	-0.434	-0.563	-0.490	-0.516	0.192	0.727	
**PB**	3.97	0.87	0.600	0.591	0.588	0.624	-0.202	-0.398	0.878

### Structural equation modeling

**Fig 5:**
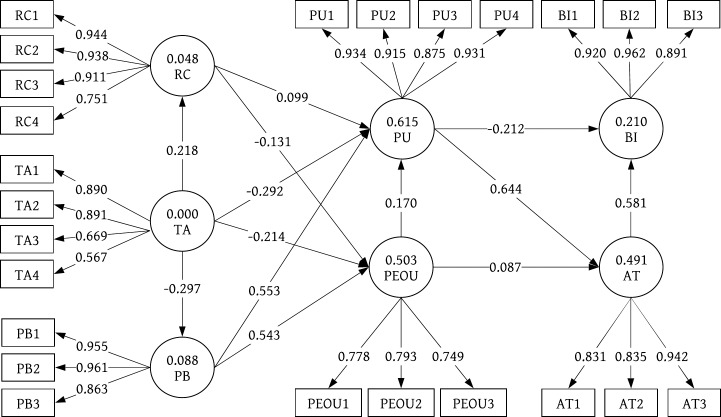
Path model of 31 older adults with cardiovascular disease.

**Fig 6:**
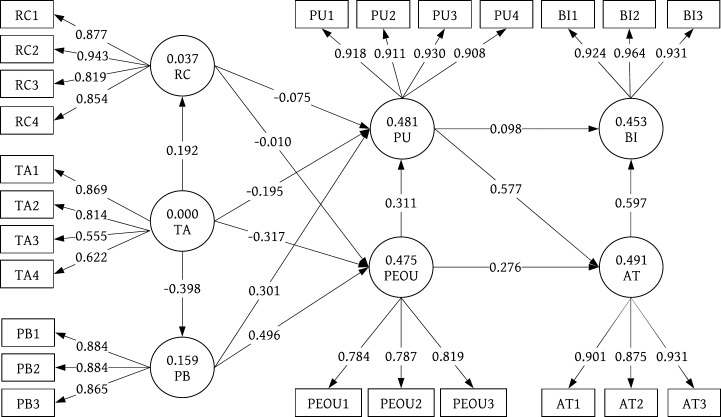
Path model of 81 older adults in general.

Table 6 presents the results of the structural model of both groups of users. The PLS-SEM analysis results (analyzed with Smart PLS 3.0) are shown in Fig 5 (older adults with cardiovascular disease) and Fig 6 (older adults in general). Regarding the relationship between TA and the other constructs, i.e., PU, PEOU, RC, and PB (H1 –H4), only two significant results were found: technology anxiety was negatively associated with the perceived ease of use of smart clothing among older adults in general (H2b β = -.317, p < .001), and technology anxiety was negatively associated with the perceived ubiquity of smart clothing among older adults in general (H4b β = -.398, p < .001). No significant associations were observed between PR and the other constructs, i.e., PU and PEOU (H5-H6), in either group of participants. Regarding the relationship between PB and the other constructs, i.e., PU and PEOU (H7-H8), there were significant results in both groups of participants. Perceived ubiquity was positively associated with the perceived usefulness of smart clothing among older adults with cardiovascular disease (H7a β = .567, p < .05) and older adults in general (H7b β = .301, p < .05). Perceived ubiquity was also positively associated with the perceived ease of use of smart clothing among older adults with cardiovascular disease (H8a β = .543, p < .001) and older adults in general (H8b β = .496, p < .05). There was also a significant relationship between PU and AI (H9). Perceived usefulness was positively associated with attitude in both user groups (H9a β = .644, p < .001; H9b β = .577, p < .05). However, no significant associations were observed between PU and BI (H10) in either user group. Regarding the relationship between PEOU and the other constructs, i.e., AT and PU, significant results were found only among the older adults in general (H11b β = .276, p < .01; H12b β = .311, p < .05). No significant results were found among the older adults with cardiovascular disease. Similarly, a relationship between AT and BI was found only among the older adults in general (H13b β = .597, p < .001). Again, no significant result was found among the older adults with cardiovascular disease. To further validate the interaction effect, we estimated the effect size (f2) by comparing the value of the R Square [59] and the Adjusted R Square value between the main and interaction effects. The size of the interaction was 0.173, which is a medium to large effect [60]. Hence, we found significant interaction effects supporting hypotheses H1a, H7a, H8a, H9a and H12a.

**Table 6:** Summary of the hypothesis results.

	IV	→	DV	Standard Deviation	T-value	p-value	Support
**H1a**	TA	**→**	PU	0.184	1.590		No
**H1b**	TA	**→**	PU	0.109	1.791		No
**H2a**	TA	**→**	PEOU	0.227	0.940		No
**H2b**	TA	**→**	PEOU	0.082	3.867	***	Yes
**H3a**	TA	**→**	RC	0.270	0.808		No
**H3b**	TA	**→**	RC	0.153	1.255		No
**H4a**	TA	**→**	PB	0.236	1.258		No
**H4b**	TA	**→**	PB	0.100	3.987	***	Yes
**H5a**	RC	**→**	PU	0.161	0.615		No
**H5b**	RC	**→**	PU	0.078	0.967		No
**H6a**	RC	**→**	PEOU	0.177	0.741		No
**H6b**	RC	**→**	PEOU	0.082	0.123		No
**H7a**	PB	**→**	PU	0.196	2.826	*	Yes
**H7b**	PB	**→**	PU	0.131	2.301	*	Yes
**H8a**	PB	**→**	PEOU	0.198	2.735	*	Yes
**H8b**	PB	**→**	PEOU	0.088	5.656	***	Yes
**H9a**	PU	**→**	AT	0.225	2.857	*	Yes
**H9b**	PU	**→**	AT	0.106	5.433	***	Yes
**H10a**	PU	**→**	BI	0.291	1.055		No
**H10b**	PU	**→**	BI	0.122	0.799		No
**H11a**	PEOU	**→**	AT	0.185	0.471		No
**H11b**	PEOU	→	AT	0.100	2.758	**	Yes
**H12a**	PEOU	**→**	PU	0.194	0.880		No
**H12b**	PEOU	**→**	PU	0.137	2.262	*	Yes
**H13a**	AT	**→**	BI	0.334	1.739		No
**H13b**	AT	**→**	BI	0.112	5.338	***	Yes

Note

* p < 0.05.

** p < 0.01.

*** p < 0.001.

In summary, our data analysis provides support for the following hypotheses: H2b (TA **→** PEOU), H4b (TA **→** PB), H7a&b (PB **→** PU), H8a&b (PB **→** PEOU), H9a&b (PU **→** AI), H11b (PEOU **→** AT), H12b (PEOU **→** PU), and H13b (AT **→** BI).

Henseler et al. [61] proposed that the standardized root mean square residual (SRMR) is a goodness-of-fit measure for PLS-SEM, and a value less than 0.1 indicates a good fit (SmartPLS 3-Model Fit, 2017). The fit indicators of the model were acceptable according to the following suggested criterion: the SRMR of the model of the older adults with cardiovascular disease was 0.128 (Saturated Model) and 0.186 (Estimated Model). Thus, the data fit the measurement model. The SRMR of the model of older adults in general was 0.088 (Saturated Model) and 0.106 (Estimated Model). Thus, the data fit the measurement model.

## Discussion

With increasing life expectancy, many developed countries have become aging societies. As people tend to live longer, older adults are more likely to develop chronic disease, such as cardiovascular disease. Given the rapid growth of technology, increasing attention has been paid to the research and development of wearable technologies in the healthcare domain. Studies have also assessed users’ perceptions of wearable technologies such as smartwatches and smartphones. However, previous products have mostly been designed based on the latest techniques or specific characteristics of patients with chronic disease. Because there is a high correlation between age and chronic disease, we aimed to understand the willingness to use and perception of using smart clothing to detect heart conditions among two groups of potential users (older adults with chronic disease and older adults in general).

In this study, we recruited 50 healthy older adults residing in a retirement community for seniors and 31 hospital outpatients who had either heart failure with a left ventricular ejection fraction <40% or severe valvular heart disease to examine their perceptions of the use of a smart clothing system. After we presented a video, an actual smart vest, and an operation scenario to the participants, we asked the participants to complete an extended technology acceptance survey. For the data analysis, we labeled the participants as older adults with cardiovascular disease (31 participants) and older adults in general (81 participants, including the 31 older adults with cardiovascular disease and 50 older adults residing in the retirement community). Regarding the older adults with cardiovascular disease, among the 13 proposed hypotheses, 3 hypotheses were supported and 10 hypotheses were invalid. Regarding the older adults in general, 8 hypotheses were supported and 5 hypotheses were invalid.

We hypothesized that technology anxiety would negatively influence the targeted users’ perception of the usefulness of smart clothing [38], perception of the ease of use of smart clothes [17], resistance to change, and perceived ubiquity. However, the results of our study only showed significant relationships between technology anxiety and perceived ease of use and perceived ubiquity among older adults in general. In both groups of users, especially cardiovascular patients, these adults value the functionality and utility of smart clothing to constantly detect their heart conditions; therefore, technology anxiety did not affect the participants’ perception of the product’s usefulness and resistance to change. However, because older adults are less familiar with wearable devices, technology anxiety toward smart clothing could negatively affect older adults’ perceived ease of use and perceived ubiquity of smart clothing [21]. Many older adults were highly concerned about the fabric of the smart clothing. Some elderly adults stated that they were afraid that their skin would be allergic to the fabric. For instance, participants were not in favor of wearing the smart clothing outside during the daytime due to fear that the fabric was not sweatproof and may cause allergic reactions to their skin. Due to their anxious feeling toward the fabric, these older adults were less likely to wear smart clothing for a long period of time, thus reducing the flexibility and ubiquity of smart clothing. Many participants stated that they preferred cotton fabric because it is perceived to have higher breathability and to be more absorbent, which may be a possible solution to reduce their anxiety and increase their willingness to wear the smart vest.

Regarding the hypotheses related to the construct of resistance to change, none of the hypotheses were supported. In both groups of participants, negative relationships were not found between resistance to change and perceived ease of use and usefulness. There are two possible explanations for these results. First, many participants expressed difficulty and confusion in understanding the semantic meaning of the questionnaire items regarding resistance to change. This misunderstanding may have affected how the participants perceived and interpreted the concept of “resistance to change”. Another explanation is that the participants did not actually experience the use of smart clothing in their daily lives. Hence, it could be difficult for them to fathom how wearing smart clothing could change the way they managed their health condition.

Based on the hypothesized model, our results indicate that perceived ubiquity is a construct that can affect the targeted participants’ perception of the smart clothing system. Similar to previous research [30, 62], patients with cardiovascular disease found that the smart clothes were easier to use due to mobility. Previously, participants had to either be at home or in the clinic and had to use medical devices to measure their heart conditions. However, with the development of mobile health technologies, patients can currently wear smart clothes to detect and manage their heart conditions ubiquitously and easily, which increases the utility value of the product. This situation also occurs among the elderly. Hence, perceived ubiquity is a vital construct that affects participants’ perceived ease of use and usefulness of wearing smart clothes.

Regarding the original TAM, among the 5 remaining hypotheses regarding PU, PEOU, AT, and BI, the relationship between PU and AT was supported in both user groups. Both groups of participants found the smart clothing system to be useful, which increased their positive attitude toward wearing this device. However, perceived usefulness did not directly impact either group of participants’ behavioral intention to actually wear smart clothing. This insignificant result may be explained by the limitation of the study. The participants were only introduced to the product for 30 minutes. They did not have prior knowledge or experience with smart clothing, which may have caused them to have lower trust toward the product. As past research has shown, users have the expectation that technology can produce accurate and reliable outcomes. Prior to using a technology, if users have lower trust toward the product, this could negatively affect users’ intention to use it [63]. As a few participants explained, they were unsure of the smart clothing’s reliability and accuracy in detecting their heart condition. Another possible explanation is that the recruited participants were all potential users of the smart vest. A previous study found that the TAM results differed between potential users and actual users of a given technology [64]. For instance, in the study by Lunney, Cunningham (29), 66% of the participants had used or were using wearable fitness technologies. Thus, among these participants, PU was positively related to BI. With regard to the relationships between PEOU and AT and PU, significant results were found only among the older adults in general. In this group, due to the ease of use of the smart clothing system, the participants believed that the product was more useful and had increased intentions to use it. This result was also observed with regard to AT and BI, for which the relationship was found only among the older adults in general. Interestingly, based on the model of older adults, the effect of PU on BI is likely to be indirect rather than direct. PU may increase older adults’ attitudes toward smart clothing and then positively affect their behavioral intention to use smart clothing. Based on the results of the model of older adults in general, most results regarding the relationships among PU, PEOU, AT, and BI aligned with previous studies [17, 28, 29]. However, because the smart clothing was designed as a medical product, surprisingly, only the relationship between PU and AT was valid among older adults with cardiovascular disease. Further research is needed to enhance our understanding of the causes of the other unsupported findings.

Overall, the results of the comparison of the models of older adults with cardiovascular disease and older adults in general indicate more significant results among the older adults in general. Because the population is aging worldwide, it is crucial to utilize integrated technologies in various fields to improve the management of older adults’ physical and mental health. Currently, however, many medical products merely focus on the latest technique while neglecting the needs of users, especially older adults, in the design process. It is essential to design medical management products from the perspective of user-centered system design [65]. As Norman emphasized, it is also crucial to involve actual users as a central part of the product development process, ideally in the context of the product’s use. Understanding older adults’ living habits and their needs in all aspects can facilitate the useful design of medical technologies, support older adults’ living conditions in various ways, and decrease older adults’ anxiety toward medical technologies. The results of our study confirm that technology anxiety and perceived ubiquity are two significant factors that need to be considered in the design of wearable medical products, such as smart clothing, for older adults.

## Conclusion

In this study, we extended the TAM and investigated perceptions of a smart clothing system among older adults with cardiovascular disease and older adults in general. The purpose of the design of the smart clothing system is to assist cardiac patients and older adults with healthcare management and enhance their quality of life. We confirmed that perceived ubiquity is a crucial construct that can affect both groups of participants’ perceived ease of use and usefulness of wearable cardiac sensing technology, such as smart clothes. The effect of technology anxiety could negatively affect perceived ease of use and perceived ubiquity among older adults due to their unfamiliarity with wearable technologies. There were no significant relationships between TA and the other constructs among the older adults with cardiovascular disease. In addition, no significant results were found regarding resistance to change in both user groups. Regarding the older adults in general, due to the ease of use of smart clothing, the participants found the smart clothing to be useful. Furthermore, the perceived usefulness of the smart clothing system was positively correlated with older adults’ attitudes toward the system. A positive correlation was also found between attitude and behavioral intention.

The results of our hypothesized model contribute to the original TAM and existing research on users’ perceptions of wearable cardiac sensing technologies. Few studies have examined older adults’ perceptions of wearable technology in healthcare or the smart clothing system. As a result, the findings of this study provide insights for the field of wearable health care sensing technology and could potentially be generalized to wearable healthcare sensing technologies that are specifically designed for medical problems.

In terms of practical implications for designing smart clothing or other medical products specifically for a chronic disease that is highly correlated with aging, our study shows that it is important not only to focus on patients’ needs but also to consider older adults in general as potential users and to consider their perceptions. Reducing users’ technology anxiety, such as by considering users’ fabric preference, and increasing the product’s ubiquity can increase users’ PU and PEOU of smart clothing, which can improve users’ attitude and ultimately increase their behavioral intention.

Due to the methodological design, the study has several limitations. First, in the process of designing a product, providing scenarios to potential users is a beneficial and frequently used method to acquire an initial understanding of users’ perceptions of a product. However, operation scenarios may not allow users the opportunity to actually test the product. Therefore, in this study, the participants were not able to fully express their thoughts regarding the measurement of technology anxiety and perceived ease of use. In future studies, an examination of users’ perceptions is suggested after they actually use a smart clothing system for several days or weeks. Second, to understand potential users’ perception of smart clothing, we recruited participants from two separate sources, outpatients with cardiovascular disease and healthy older adults residing in a retirement community. However, because our study population was specific to cardiovascular patients, it was difficult to recruit a large number of participants. Even among the outpatients who met the criteria, not all patients were willing to participate in the study. Thus, the sample of older adults with cardiovascular disease was small. Finally, this study focused only on older adults. To fully understand the effect of age differences on potential users’ perceptions of smart clothing, future studies can recruit younger adults (between the ages of 20 and 65) as participants and compare the results with our study.

## Supporting information

S1 File TAM questionnaire. (PDF)

S2 File TAM Data. (XLSX)
