# Supplementary material for: Technology anxiety and resistance to change behavioral study of a wearable cardiac warming system using an extended TAM for older adults
Source: PLoS One. 2020 Jan 13;15(1):e0227270. doi: 10.1371/journal.pone.0227270 (PMC6957166; doi:10.1371/journal.pone.0227270)
Supplement: S1 File — (PDF) [file pone.0227270.s001.pdf]

Researcher: Date:

Section One: Background Information

[1] Gender Male Female

[2] Age: _______________ years old

[3] Educational Level:

 Elementary School Junior High Senior High Bachelor’s Degree

 Graduate Degree

Section Two: TAM Questionnaire

		Strongly disagree	Disagree	Neutral	Agree	Strongly Agree
RC1	I don’t want the smart clothing to change the way I deal with health-related problems	1	2	3	4	5
RC2	I don’t want the smart clothing to change the way I keep myself healthy.	1	2	3	4	5
RC3	I don’t want the smart clothing to change the way I interact with other people.	1	2	3	4	5
RC4	Overall, I don’t want the smart clothing to change the way I currently live.	1	2	3	4	5
TA1	I feel apprehensive about using smart clothes.	1	2	3	4	5
TA2	I hesitate to use technology for fear of making mistakes I cannot correct.	1	2	3	4	5
TA3	I am afraid that the equipment may suddenly stop functioning.	1	2	3	4	5
TA4	I don't want other people to see me wearing the smart clothing.	1	2	3	4	5
PB1	The smart clothing system provides healthcare information anytime and anywhere.	1	2	3	4	5

PB2	The smart clothing system provides me with anytime-and-anywhere communication and connectivity.	1	2	3	4	5
PB3	I will use the smart clothing system very often for health purposes.	1	2	3	4	5
PEOU1	I find the smart clothing to be clear and understandable.	1	2	3	4	5
PEOU2	I find that the smart clothing does not require a lot of mental effort.	1	2	3	4	5
PEOU3	I find the smart clothing to be easy to use.	1	2	3	4	5
PU1	Using the smart clothing will improve my quality of life.	1	2	3	4	5
PU2	Using the smart clothing will make my life more convenient.	1	2	3	4	5
PU3	Using the smart clothing will make me more effective in my life.	1	2	3	4	5
PU4	Overall, I find the smart clothing to be useful in my life.	1	2	3	4	5
AT1	I think that using the smart clothing is a good idea.	1	2	3	4	5
AT2	I think that using the smart clothing is beneficial to me.	1	2	3	4	5
AT3	I have a positive perception of using the smart clothing.	1	2	3	4	5
BI1	I intend to use the smart clothing in the future.	1	2	3	4	5
BI2	I will always try to use the smart clothing in my daily life.	1	2	3	4	5
BI3	I plan to use the smart clothing frequently.	1	2	3	4	5
